# Functional and Structural Changes in Patients with Spinal Muscular Atrophy Treated in Poland during 12-Month Follow-Up: A Prospective Cohort Study

**DOI:** 10.3390/jcm13144232

**Published:** 2024-07-19

**Authors:** Aleksandra Bieniaszewska, Magdalena Sobieska, Ewa Gajewska

**Affiliations:** 1 Department of Developmental Neurology, Poznan University of Medical Sciences, 60-355 Poznan, Poland; ewagajewska1011@gmail.com; 2 Doctoral School, Poznan University of Medical Sciences, 60-812 Poznan, Poland; 3 Department of Rehabilitation and Physiotherapy, Poznan University of Medical Sciences, 61-545 Poznan, Poland; msobieska@ump.edu.pl

**Keywords:** spinal muscular atrophy, functional assessment, structural assessment, ranges of motion, joint contractures

## Abstract

**Background:** In recent years, rapid advances in diagnosis and treatment have been observed in spinal muscular atrophy (SMA) patients. The introduction of modern therapies and screening tests has significantly changed the clinical picture of the disease. The previous classification has, therefore, been replaced by new phenotypes: non-sitters, sitters, and walkers, defined by the patient’s functional level. However, despite the change in the clinical picture of the disease, patients still suffer from accompanying structural disorders such as scoliosis or joint contractures. Their presence also significantly affects the acquisition of subsequent motor skills. Due to this, monitoring structural changes and ensuring therapists are aware of improvements or declines in patient functionality are essential components of clinical practice. This study aims to compare the assessment of structural and functional changes after a 12-month follow-up in SMA patients who have already experienced the effects of the disease and are now receiving modern therapy. **Methods:** We present a study of 34 SMA patients being treated with modern therapies and tested twice 12 months apart. The participants were tested using structural measurements and validated scales such as The Children’s Hospital of Philadelphia Infant Test of Neuromuscular Disorders (CHOP-INTEND) and Hammersmith Functional Motor Scale–Expanded (HFMSE). **Results:** During the 12-month follow-up, patients showed deteriorating, non-statistically significant structural changes. We also proved that patients showed a trend toward functional improvement. Analyzing the individual scale items, we distinguished which participants obtained the maximum score for a given parameter and no longer had an opportunity to improve during the second examination. **Conclusions:** Our study proved that most patients improved overall motor function. The examination of structural measurements should become a standard in the evaluation of SMA patients.

## 1. Introduction

Spinal muscular atrophy (SMA) is one of the most prevalent neuromuscular disorders inherited in an autosomal recessive manner [1]. Mutation or deletion of the motoneuron survival gene (SMN1) is the cause in 95% of cases, resulting in insufficient production of SMN protein [2,3]. In its natural, untreated course, the disease manifests as progressive muscular atrophy of the extremities and trunk, resulting in gradual loss of physical function, leading to disability and, in the most severe cases, death [4].

In recent years, rapid advances in diagnosis and treatment have been observed in SMA patients [4,5,6]. The US Food and Drug Administration (FDA) and the European Medicines Agency (EMA) have approved three medicines to alter the course of SMA [7]. Spinraza (Nusinersen), Zolgensma (Onasemnogen abeparwowek, gene therapy), and Evrysdi (Risdiplam) significantly improved the quality of function of patients, who, instead of gradual progression of the disease, not only began to show improvement in function but also presented the ability to reach new milestones [2,3,4,8]. All three drugs have been used in Poland under a government-funded drug program that granted reimbursement for SMA patients (Nusinersen since 1 January 2019; Risdiplam and Zolgensma since 1 September 2022) [9,10]. Since then, all children in Poland have been tested at birth, and treatment is immediately implemented. However, children diagnosed with SMA before the drug was approved received this treatment later. Thus, they may have already partially developed changes caused by the natural, untreated course of the disease. A newborn screening program has been implemented in Poland since March 2022. This program has allowed for the screening of 382,000 newborns, and has detected SMA in 53 of them (as of 30 November 2022). Thus, Poland has joined countries such as Norway, Germany, and Belgium as leaders in European SMA treatment [9,11].

Modern therapeutic approaches and the introduction of screening tests have significantly changed the clinical picture of the disease. The previously used division into different subtypes is no longer valid. As a result of the rapid application of treatment, children defined by type I who previously would never have the ability to sit unsupported now sometimes present higher skills such as standing and walking [10,12,13,14]. The previous classification has, therefore, been replaced by new phenotypes, such as non-sitters, sitters, and walkers, which are defined by the patient’s functional level [4,5,15]. Nevertheless, patients continue to experience associated structural problems such as scoliosis or joint contractures to a lesser extent, affecting the upper limbs rather than the lower extremities, even when the disease’s clinical presentation has changed. This problem mainly affects patients who were not treated immediately after birth. We must remember that at the time of the introduction of treatment in Poland, the program was extended not only to children from genetic screening for SMA but also to older children in whom the natural course of the disease has led to a weakening of muscle strength, resulting in the formation of structural changes. Their presence also significantly affects the acquisition of subsequent motor skills [16,17,18,19,20].

Thus, this study aims to compare the assessment of structural and functional changes after 12-month follow-up in SMA patients who have already experienced the effects of the disease and are now receiving modern therapy.

## 2. Materials and Methods

The research is a continuation of our previous observations [10,21]. The data were collected between May 2020 and February 2023.

### 2.1. Patient Sample

This study involved forty-four patients recruited from the Department of Developmental Neurology at Poznan University of Medical Science, Poland. The study’s inclusion criteria consisted of a genetically confirmed diagnosis of SMA and informed consent from parents or guardians. Exclusion criteria included other genetic or metabolic diseases affecting children’s motor function, children with a diagnosis obtained by screening, and lack of informed consent from parents or guardians.

During the follow-up, ten patients were excluded from the research. Two patients discontinued observation due to COVID-19 quarantine, two changed their residence, and one was aged out and assigned to a different department within the hospital. Five patients were also excluded from the study because of changes in treatment type during follow-up.

Thirty-four patients diagnosed with SMA aged 2 to 20 years were included in the sample. All of them were diagnosed later than infancy and started treatment after the first clinical symptoms appeared. Twenty of the patients were females and fourteen were males. All participants underwent pharmacological treatment with Nusinersen (15 patients) and Risdiplam (19 patients). Most of the patients were treated before the age of 6, and they received the drug at an average of 71 months after diagnosis.

Each patient participated in rehabilitation 1–3 times a week according to the International Commission on Standards of Care for Spinal Muscular Atrophy guidelines published in 2018 [12,13,14]. Nine patients with SMA type 1, eighteen with SMA type 2, and seven with SMA type 3 were included in the study. This categorization was based on the previously used SMA division. Children whose first symptoms occurred before they reached six months of age were considered to have the most severe type 1 SMA. These patients did not live to be two years old and never achieved the ability to sit up independently. Patients with SMA2 were children who were diagnosed between 6 and 18 months of age, and the highest function they presented was unsupported sitting. Patients with SMA3, on the other hand, were considered to be patients with a diagnosis after 18 months of age who had achieved walking function. SMA0, in which the first symptoms began as early as fetal life, and SMA4, involving adult patients, were also clinically defined [1,4].

Nevertheless, a new functional division is currently used to describe SMA individuals. Consequently, four kids belonged to the walkers’ group, seven to the non-sitters’ group, and twenty-three to the sitters’ group. Table 1 presents all clinical data concerning the study group.

### 2.2. Study Procedures and Outcomes

The patients were examined twice during a 12-month follow-up: at the baseline and after 12 months +/− 2 weeks. The first and second examinations were always performed by the same two investigators trained in analyzing functional scales and structural measurements, with extensive clinical experience in pediatric physiotherapy and with patients with SMA. The study consisted of evaluating the structural and functional changes in SMA patients. The structural measurements protocol included the range of motion of upper and lower extremities and trunk parameters for early detection of musculoskeletal changes predisposing to scoliosis formation. The functional assessment was based on the functional scales. A detailed description of the methodology is presented below.

### 2.3. Structural Parameters

During the examination, structural characteristics in the trunk and extremities were assessed. Within the trunk, the following parameters were evaluated. The CR parameter presents the extent of cervical spine rotation. The SATR measures the angle of the upper (SATR-U) and lower (SATR-L) trunk rotation. The HE assesses the hip extension and the PO determines the value of the pelvic tilt angle. The SATR and PO parameters were evaluated using a scoliometer and CR and HE were examined with a plurimeter (Rippstain Inclinometer) [10,19,20,21]. The side length trunk difference (SLTD) was measured while the subject was seated. The posterior superior iliac spine and inferior angle of the scapula were marked anatomically as recognized surface landmarks. A tape measure was used to measure the distance on the same side between these two spots [19].

A goniometer measured the upper and lower extremities’ neutral position and range of motion. The value of joint contracture was determined if the child could not reach a neutral position. The following parameters were included: in the upper limb, arm flexion (AF) and abduction (AB), elbow flexion (EF), and forearm supination (FS). In the lower limb, flexion of the hip (HF) and knee (KF) and ankle dorsiflexion (AF) [16].

### 2.4. Functional Assessment

**Figure 1:**
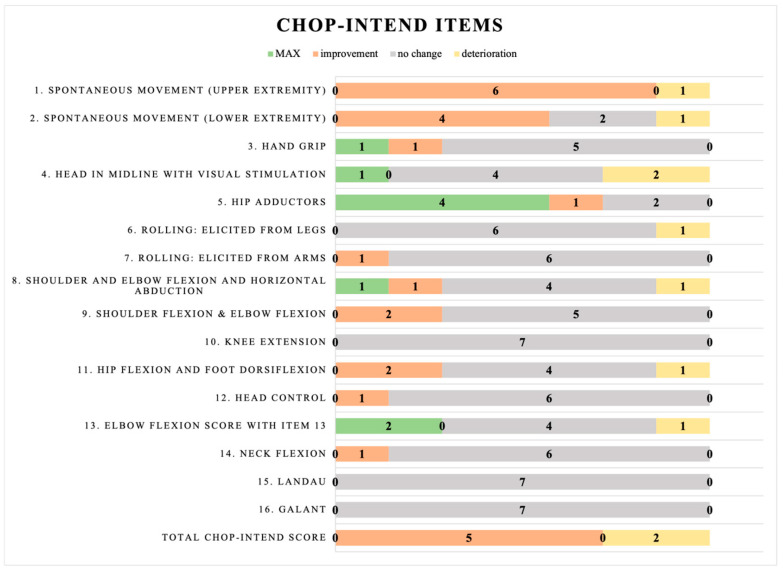
Changes for the non-sitters’ group in individual CHOP-INTEND scale items.

**Figure 2:**
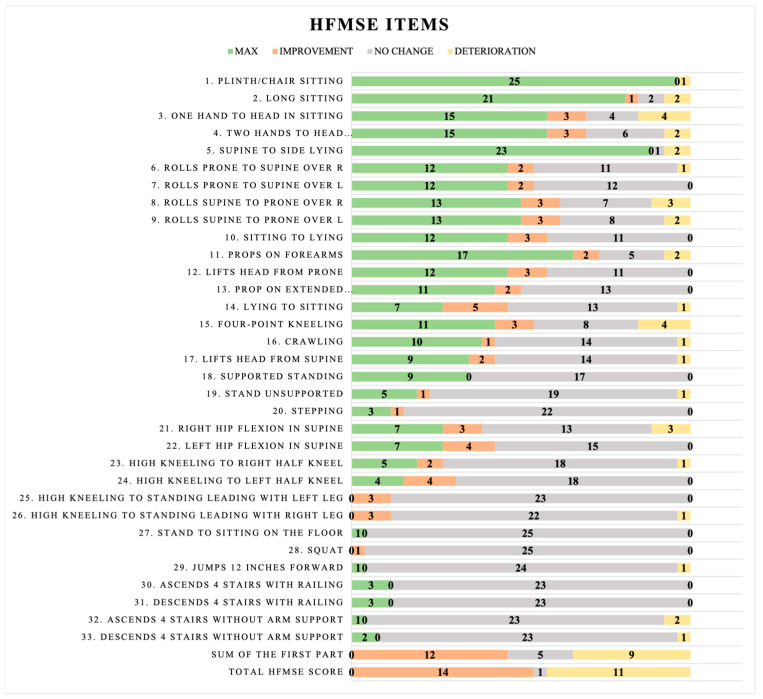
Changes in individual HFMSE scale items for sitters and walkers.

Gross motor function was evaluated using The Children’s Hospital of Philadelphia Infant Test of Neuromuscular Disorders (CHOP-INTEND) and The Hammersmith Functional Motor Scale–Expanded (HFMSE). Both of the scales were previously validated [22,23,24]. Patients with the most severe type of SMA, who are weak patients who cannot maintain a sitting position, were examined using the CHOP-INTEND scale. The scale assessed 16 activities, including head control, spontaneous movement of upper and lower limbs, shoulder, elbow, and hip flexion, and Landau and Galant reflexes. Every task is assigned a score between 0 and 4 points, with a maximum of 64 possible points [22]. All items of the scale are presented in Figure 1. The other, the HFMSE scale, was used for children who at least demonstrated the function of unsupported sitting. The scale comprises the basic version (20 items) and the supplementary module (13 items). The tasks evaluated are sitting, rolling, propping on forearms and extended arms, changing positions from sitting to standing, squatting, or climbing stairs. Every question has a possible score range of 0 to 2, where two indicates a task was finished correctly, 1 indicates that the task was performed with compensation, and 0 indicates the task was not completed. The highest sum of points that may be scored is 66 [23,24]. All items of the scale are presented in Figure 2. Our previous study published detailed characteristics of functional scales and structural measurements [10,21].

The Bioethical Committee of Poznan University of Medical Sciences approved the study (no. 1035/19).

### 2.5. Statistical Analysis

All statistical analyses were performed using StatSoft’s (Krakow, Poland) Statistica 12.2 software. The results were presented as the median and quartiles [Me (Q25–Q75)] for both interval variables (as the data distribution differed from normal) and for ordinal variables. The non-parametric Mann–Whitney U test was used to calculate the differences between the investigated groups, and the sign test was used to calculate the time difference. The correlations between the variables were calculated using the non-parametric Spearman’s test, and the rho value was given. For all the tests used, a *p* < 0.05 was regarded as significant.

## 3. Results

Due to the significant differences in the patient’s functional level, all results were described according to new phenotypes classification, which divided patients into groups of non-sitters, sitters, and walkers.

### 3.1. Structural Changes in Trunk and Lower-Extremity Parameters

After a preliminary analysis of the results, no significant differences were observed between the presented upper extremity range values and the accepted norms, so the presentation of these data was abandoned. For the walkers’ group, which consisted of four children only, the min−max values are shown, as well as the number of patients who showed improvement, no change, and deterioration because of the walker’s group’s relatively small size (n = 4). Table 2 presented an extensive description of the trunk parameter results.

When analyzing the trunk parameters in the no-sitters group, an improvement was observed in one value—left hip extension (HE-L). This change affected four patients. Function deterioration was seen in four patients, and parameters such as right cervical rotation (CR-R) and right hip extension (HE-R) were involved. No change included five patients and was seen in the values of the trunk rotation (SATR-U and SATR-L). Due to the inability of patients to sit up independently, the pelvic oblique (PO) and side length trunk difference (SLTD) were not analyzed in this group.

In the sitters’ group, most patients showed no changes in parameters such as trunk rotation (SATR-U and SATR-L), pelvic oblique (PO), or side length trunk difference (SLTD). Improvement was seen in the left cervical rotation (CR-L)—13 patients, right cervical rotation (CR-R)—12 patients, and left hip extension (HE-L)—12 patients. A slight deterioration was observed in the parameter of the right hip extension (HE-R). The negative change affected ten patients, while improvement in this parameter was noted in nine children.

In the walkers’ group, the most improvement was seen in parameters such as right cervical rotation (CR-R) and the right and left hip extension (HE-R, HE-L). No change prevailed for the rest of the parameters.

The 12-month difference in ranges of motion in the lower extremities and changes in neutral positions indicating worsening or improvement of joint contractures were studied. The results are presented in Table 3.

In the non-sitters’ group, all ranges of motion showed no change in most patients at the 12-month follow-up. However, all neutral positions determining the value of contracture worsened.

For the sitters’ group, the final ranges of motion of the right hip flexion (HF-R), flexion of both knees (KF-R, KF-L), and dorsiflexion of the right foot (AD-R) improved. Aside from the right knee flexion parameter (KF-R), which affected 11 patients, no other changes in joint contractures were observed in this group.

In the walkers’ group, neither the range of motion nor neutral positions changed.

### 3.2. Analysis of Functional Changes Based on the Motor Scales

Overall scores, as well as individual items in functional scales, were analyzed. Patients were tested using the tools adapted to their functional level. The CHOP-INTEND scale was used to test participants in the non-sitters’ group, and the HFMSE scale was used to assess patients in the sitters and walkers’ group.

Table 4 presents a comparison between the first and second examination outcomes. The walkers’ group did not have its median, Q25–Q75, or statistical significance examined because of the small group size.

In the non-sitters’ group, five patients showed improvement and two showed deterioration in scale scores between the first and the second examinations. The change was not statistically significant.

Considering the sitters’ group, the median scale scores decreased in the HFMSE Part 1 and HFMSE Total sequentially from 22 to 21 in the HFMSE Part 1 and from 24 to 23 points in the HFMSE Total. However, analyzing the patients’ results, most patients showed improvement: 11 in HFMSE Part 1 and 12 in the HFMSE Total. These changes were not statistically significant, either.

In the walkers’ group, three patients showed no change in the scores of the HFMSE Part 1. Analyzing the HFMSE Total score, two patients showed improvement and another two presented deteriorations in function.

### 3.3. Difference in Individual Scale Items

The results of the CHOP-INTEND scale for non-sitters’ and the HFMSE scale for sitters and walkers are shown in Figure 1 and Figure 2, respectively. The items were divided into MAX, improvement, no change, or deterioration. The MAX subgroup included children who could not show improvement (as they reached MAX in the first examination), and they did not deteriorate, either.

Five out of seven patients in the non-sitters’ group showed improvements according to the CHOP-INTEND scale during the follow-up. None of the patients achieved the highest possible score. Considering individual items, the most significant changes were observed in parameters of spontaneous movement of upper and lower extremities, achieving improvement in six and four patients, respectively. The only parameter with a dominance of the max value was hip adductors, which included four patients. No changes were observed in the other scale items.

The results for the HFMSE scale are presented in Figure 2.

During the 12-month analysis, 14 patients improved on the HFMSE scale, while nine showed deterioration and five showed no change in function. Most parameters showed no change. In the first part of the scale, the lack of change was due to obtaining the maximum score in an individual item and receiving the so-called ceiling effect. However, the situation was different considering the second part of the scale and more complex functional tasks. The lack of change was due to the lack of skill progression and, thus, the inability to obtain higher scores on the scale.

The study also analyzed correlations between structural parameters and their effects on motor function. The statistical significance of the above parameters was not shown. The only significant correlation was for hip extension (HE) during the second examination. For the left hip (HE-L), it was rho = 0.42 for HFMSE Part 1 and rho = 0.39 for HFMSE Total. For the right hip (HE-R), the correlation coefficient was rho = 0.52 for HFMSE Part 1 and rho-0.51 for HFMSE Total.

## 4. Discussion

The main issue of this study was to compare the assessment of structural and functional changes after 12-month follow-up in SMA patients who have already experienced the effects of the disease and are now receiving modern therapy. This issue still needs to be addressed. Functional scales that are used to evaluate children should take such parameters into account. It was essential to obtain answers as to whether modern therapies influence the change in structure and, therefore, whether the change in structure influences the change in motor function. From a clinical point of view, we wanted to determine whether our patients are deteriorating in function or losing it precisely because of increasing structural changes.

The group described in the present study illustrates the transitional period between the times when a diagnosis of SMA meant a continuous deterioration of motor function, not infrequently leading to extreme functional impairment, and the current situation in which early diagnosis and immediate implementation of treatment that levels the deficits is possible. In addition, we are talking about children who have experienced the natural, untreated course of the disease, in whom the function deteriorated due to weakening muscles, which might have caused structural changes.

Therefore, it is necessary to consider that this group is strongly heterogeneous primarily due to the wide range of ages and the period between diagnosing the disease and implementing modern treatment. However, SMA is a rare disease, and all these patients come from one region of Poland and are under the surveillance of one clinic, one of the few in Poland specializing in rare diseases such as SMA. As a result of too long a period passing between the diagnosis and the treatment, some children experience motor deficits, and some develop adverse structural abnormalities (contractures, trunk asymmetry, and scoliosis). It is gratifying to note that most patients have ceased their motor deterioration. After a one-year follow-up, there was no statistically significant deterioration of structural disorders and some children even exhibited improved motor function. This may indicate that modern therapy has stopped the progressive weakening of muscle strength and led to the maintenance and even improvement of the functional level.

The introduction of treatment in Poland (started in 2019 and expanded in 2022) and its impact on the functional and structural changes in patients has made the SMA children a fascinating group from a clinical point of view [4,5,9,15]. However, the literature still indicates the need for further follow-up of patients with particular attention to the formation of structural changes and joint contractures. Nicolau and co-authors noted the impact of weight gain and worsening contractures on the rapid function deterioration in SMA patients. In contrast, Mirea and co-authors emphasized the need to maintain the range of motion and increase muscle strength as a prerequisite for achieving new functions [25,26]. A review by Barnello and co-authors mentioned that individuals aged 6 to 15 years old were particularly vulnerable to developing joint contractures or scoliosis progression, which negatively affected functional outcomes and can influence patients’ therapeutic response [27]. Our clinical observations showed no statistically significant deteriorating structural changes in treated patients. The only deteriorating parameters were neutral positions for hip extension and hip flexion, particularly in the sitters’ and non-sitters’ groups. The deterioration was probably due to stronger contractors than extensor muscles. We suppose that under treatment, the muscles ceased to weaken, but at the same time, these children do not use both muscle groups as they sit or are seated. The walkers did not experience this deterioration, probably because they can move and use both contractor and extensor muscles in balance. These changes, however, did not worsen enough to affect function, suggesting that treatment stopped the progressive atrophy.

The patients we studied form a transitional group in which treatment was implemented late. The average time from obtaining the diagnosis to starting treatment was about 21 months. This information and the fact that the patients began treatment with a median age of 5 years explains the occurrence of several structural and functional alterations in some patients, which was particularly evident in the non-sitters’ group; in our study, this represented the late and the most severe course of the disease. These patients were older than others (median age of 12 years), and their treatment was implemented much later (122 months, whereas in the sitters’ group, it was 65 months).

In our study, no deterioration of motor function was observed, as might have been expected before the period of treatment implementation. These results are cause for great optimism. In addition, the maintenance of functional status was undoubtedly influenced by targeted rehabilitation conducted in Poland, according to the guidelines of the International Committee for Standards of Care for Spinal Muscular [12,13,14]. In our opinion, examining structural changes should become a standard in evaluating SMA patients. Their assessment will enable the correct therapy adjustment to the patient’s condition and prevent the formation of contractures and unfavorable compensations affecting the impaired achievement of subsequent functions. Also, measuring structural changes as a management standard in SMA will allow early detection of possible changes and their ongoing correction, which is particularly important for children diagnosed with screening. Similarly, Trenkle and co-authors pointed out this issue in their paper, discussing the need to increase the frequency and duration of physiotherapy in patients receiving drug therapy [15].

Another examined issue was a functional analysis using validated tests. We proved that our patients showed a trend toward functional improvement during the 12-month follow-up. Patients analyzed with the CHOP-INTEND scale, i.e., the non-sitters’ group, showed functional improvement in five of seven children studied. Analyzing the final score on the HFMSE scale in the sitters’ group, improvement was noted in 12 patients, and no change was observed in the other 2. Given that improvement and lack of deterioration are considered to represent clinical success, this is also the predominant result despite an apparent decrease in the median score from 24 to 23 points. The situation was different in the walkers’ group, where the same number of patients showed improvement and deterioration in the overall scale score. This agrees to some extent with the systematic review by Erdos and co-authors, in which the authors described that as a result of treatment with Nusinersen, gene therapy, and combination therapy, improvement in motor function was noted in patients with SMA1, and patients with SMA2 to SMA4 type, stabilization or slight improvement, but also some deterioration of function [7].

The final component of the study was to determine whether there was improvement, deterioration, or no change in individuals in the given items of the CHOP-INTEND scale and the HFMSE scale during a 12-month follow-up. It was shown that in the CHOP-INTEND scale, the parameters of spontaneous movement of the upper and lower extremities improved markedly in six and four patients, respectively. In contrast, in the Hammersmith scale analyzing sitting and walking patients, no function showed a notable improvement, and most of the analyzed items remained unchanged. Therefore, we decided to check whether the given items in the scale did not change because the patients did not show improvement or perhaps reached the maximum score (MAX) in a particular scale item already seen during the first examination. Thus, a clear dominance of the maximum score in the first part of the scale was noted, while there was no change in the second part of the HFMSE test (which contains more difficult function items). As a result of such analysis, whether the validated scales used for functional assessment of SMA are sufficient is questionable. Day has also evaluated this topic. In his publication, he described that the slight increase in the mean score of the HFMSE scale is due to the increasing difficulty of items found in the second part of the scale (such as squatting, jumping, or climbing stairs) and thus, the much lower possibility of their improvement regardless of type [28]. We need to consider whether a different assessment tool, which may be more in line with their current abilities, should be used to evaluate children diagnosed with SMA to avoid a ceiling effect on the upper extremities. We have already referred to this observation in our previous article [21].

In our study, we wanted to show how structure changes in SMA patients and whether this change affects specific functions. Introducing measurement of structural changes as a standard of management for SMA will thus allow possible changes to be caught early and corrected on an ongoing basis.

There are some limitations of the study. The main problem was the failure to include the analysis of spinal X-rays when evaluating structural changes. The second was the high heterogeneity of the group. This problem is common in rare diseases such as SMA [7,29]. The last limitation was that the study included only one center. Although our Clinic is one of the critical centers in Poland dealing with spinal muscular atrophy patients, it would be worthwhile to extend our observation to other centers in Poland and worldwide.

## 5. Conclusions

At the 12-month follow-up, no statistically significant structural changes in ranges of motion or joint contractures were noted. Our study proved that most of the patients showed improved overall motor function. It is worth emphasizing that the lack of change in individual scale items is not only due to a lack of improvement in function and failure to achieve new skills but is also due to achieving a maximum score already present at the initial follow-up. The most important conclusion is that examining structural changes should become the standard when evaluating SMA patients.

## Figures and Tables

**Table 1:** Study group characteristic.

Parameters	All (*n* = 34)	Non-Sitters (*n* = 7)	Sitters (*n* = 23)	Walkers (*n* = 4)
Sex:				
Male	14	2	11	1
Female	20	5	12	3
Age (given in years):				
Median (Q25–Q75)	8 (5–12)	12 (8–13)	7 (6–10)	9 (5–12)
SMN2 copy number:				
2 copies	8	5	3	-
3 copies	20	1	17	2
4 copies	6	1	3	2
Child’s functional level:				
Recumbent	7	7	-	-
Sits unassisted	6	-	6	-
Rolls over	7	-	7	-
Crawls	4	-	4	-
Stands with assistance	4	-	4	-
Stands unassisted	2	-	2	-
Walks with assistance	1	-	-	1
Walks unassisted	3	-	-	3
Rehabilitation intensity:				
1–3 times a week	5	1	1	3
4–6 times a week	15	3	11	1
Every day	14	3	11	-
Therapy used:				
Nusinersen	15	1	10	4
Risdiplam	19	6	13	-
Age at the start of treatment (given in years):				
median (Q25–Q75)	6 (3–11)	10 (6–12)	5 (3–9)	8 (5–11)
The duration between the start of treatment to the first examination (given in months):				
median (Q25–Q75)	22 (10–36)	15 (6–26)	30 (15–36)	30 (18–51)
The duration between the diagnosis and the start of the treatment (given in months):				
median (Q25–Q75)	72 (43–138)	122 (77–142)	65 (44–110)	101 (58–142)

**Table 2:** Results from trunk parameters during the first and second examinations.

Parameter	First Examination			Second Examination			Number of Children with Improvement/ No Change/ Deterioration		
	Median	Min–Max	Q25–Q75	Median	Min–Max	Q25–Q75	Improvement	No Change	Deterioration
Non-Sitters (*n* = 7)									
CR-L	70	40–80	50–80	70	35–80	45–80	2	2	3
CR-R	55	20–80	40–70	50	15–80	50–65	2	1	4
SATR-U	5	5–15	5–5	5	0–15	5–10	1	5	1
SATR-L	5	5–20	5–5	5	5–20	5–5	1	5	1
HE-L	−10	−30–30	−10–0	−15	−25–30	−20–0	4	2	1
HE-R	0	−20–35	−20–0	−10	−30–40	−20–10	3	0	4
PO	-	-	-	-	-	-	-	-	-
SLTD	-	-	-	-	-	-	-	-	-
Sitters (*n* = 23)									
CR-L	80	40–90	70–80	80	50–90	75–90	13	4	6
CR-R	70	30–85	60–75	70	40–90	65–80	12	6	5
SATR-U	5	0–15	0–5	5	0–15	0–5	5	14	4
SATR-L	5	0–15	0–5	5	0–15	0–5	7	13	3
HE-L	10	−60–35	−15–25	0	−45–40	−10–20	12	1	10
HE-R	5	−50–45	−10–25	−5	−45–40	−10–25	9	4	10
PO	5	0–30	5–15	10	0–30	5–15	3	13	7
SLTD	1	0–7	0–2.5	3	0–8.5	1–4	4	19	0
Walkers (*n* = 4)									
CR-L	-	70–90	-	-	70–90	-	2	0	2
CR-R	-	70–85	-	-	80–90	-	3	1	0
SATR-U	-	0–5	-	-	0–5	-	1	3	0
SATR-L	-	0–5	-	-	0–5	-	1	2	1
HE-L	-	30–45	-	-	25–35	-	3	0	1
HE-R	-	25–45	-	-	20–35	-	4	0	0
PO	-	0–10	-	-	0–10	-	1	3	0
SLTD	-	0–5	-	-	0–2	-	1	3	0

Abbreviations: CR-L and CR-R—left and right cervical rotation; SATR-U and SATR-L—upper and lower trunk rotation angle; HE-L and HE-R—left and right extension of the hip; PO—pelvic obliquity; SLTD side length trunk difference.

**Table 3:** The lower extremities’ motion range and neutral positions on the first and second examinations.

Parameter	First Examination			Second Examination			Number of Patients with Improvement/ No Change/ Deterioration		
	Median	Min–Max	Q25–Q75	Median	Min–Max	Q25–Q75	Improvement	No Change	Deterioration
Non-Sitters (*n* = 7)									
Range of motion									
HF-L	120	100–130	110–125	120	110–135	110–130	2	4	1
HF-R	115	100–125	100–125	120	100–135	100–130	3	2	2
KF-L	140	115–150	130–145	140	130–150	130–150	2	5	0
KF-R	140	120–150	130–150	140	115–155	140–155	2	2	3
AD-L	0	0	0	0	(−45)–5	0–5	2	4	1
AD-R	0	0	0	0	(−40)–10	0–5	2	4	1
Neutral position									
HF-L	30	10–50	20–40	45	30–50	30–50	0	2	5
HF-R	30	10–50	15–30	35	30–80	30–45	1	2	4
KF-L	70	40–80	50–70	60	50–80	50–80	2	2	3
KF-R	60	30–80	40–70	60	50–80	50–80	1	2	4
AD-L	−15	(−50)–0	(−40)– (−5)	−30	(−45)– (−10)	(−40)– (−10)	1	2	4
AD-R	−20	(−50)– (−10)	(−50)– (−20)	−30	(−50)– (−5)	(−40)– (−20)	2	2	3
Sitters (*n* = 23)									
Range of motion									
HF-L	125	105–140	115–130	123	105–140	115–130	8	9	6
HF-R	120	105–145	115–130	125	100–140	120–130	11	7	5
KF-L	150	120–160	140–155	150	130–160	145–160	16	6	1
KF-R	150	120–160	140–155	150	120–160	145–160	14	8	1
AD-L	0	0–10	0–10	10	0–20	0–10	10	11	2
AD-R	5	0–15	0–10	10	0–20	5–10	14	6	3
Neutral position									
HF-L	20	0–65	10–30	20	0–50	10–40	4	10	9
HF-R	15	0–55	5–20	20	0–55	10–30	2	11	10
KF-L	30	0–100	10–55	30	0–95	20–55	4	10	9
KF-R	30	0–100	10–60	30	0–100	20–50	8	4	11
AD-L	0	(−15)–0	0–0	0	(−10)–0	0–0	4	18	1
AD-R	0	(−20)–10	(−5)–0	0	0–0	0–0	6	16	1
Walkers (*n* = 4)									
Range of motion									
HF-L	-	120–140		-	120–140		0	3	1
HF-R	-	110–140		-	110–140		0	3	1
KF-L	-	145–155		-	140–155		1	2	1
KF-R	-	140–155		-	140–155		0	4	0
AD-L	-	0–15		-	0–10		1	2	1
AD-R	-	0–20		-	0–10		1	2	1
Neutral position									
HF-L	-	0–0		-	0–0		0	4	0
HF-R	-	0–0		-	0–0		0	4	0
KF-L	-	0–15		-	0–15		0	3	1
KF-R	-	0–15		-	0–15		0	3	1
AD-L	-	(−10)–0		-	(−30)–0		0	2	2
AD-R	-	(−15)–0		-	(−30)–0		0	1	3

Abbreviations: HF-L and HF-R—left and right hip flexion; KF-L and KF-R—left and right knee flexion; AD-L and AD-R—left and right ankle dorsiflexion.

**Table 4:** Comparison of functional scales between first and second examination.

Parameter	Non-Sitter: (*n* = 7)	Sitter: (*n* = 23)		Walker: (*n* = 4)	
	Chop-Intend	HFMSE Part 1	HFMSE Total	HFMSE Part 1	HFMSE Total
First Examination					
Me	15	22	24	-	-
Min−max	2–39	3–40	5–59	11–40	8–40
Q25–Q75	7.25–28.75	12–35	14–41		
Second Examination					
Me	15	21	23	-	-
Min−max	1–43	5–40	6–58	13–63	10–66
Q25–Q75	9.75–33	12–36	14–40		
Comparison between examinations					
Difference	Z = 1.26 *p* = 0.207	Z = 0.43 *p* = 0.667	Z = 0.22 *p* = 0.826	-	-
Improvement (*n*=)	5	11	12	1	2
No change (*n*=)	0	3	2	3	0
Deterioration (*n*=)	2	9	9	0	2

## Data Availability

The informed consent/assent accounts for the publication of our research data are available upon request.
